# Determinants of knowledge, attitude, and practice towards first aid among kindergarten and elementary school teachers in Gondar city, Northwest Ethiopia

**DOI:** 10.1186/s12873-021-00468-6

**Published:** 2021-06-21

**Authors:** Belayneh Shetie Workneh, Enyew Getaneh Mekonen, Mohammed Seid Ali

**Affiliations:** 1 Department of Emergency and Critical Care Nursing, School of Nursing, College of Medicine and Health Sciences, University of Gondar, Gondar, Ethiopia; 2 Department of Surgical Nursing, School of Nursing, College of Medicine and Health Sciences, University of Gondar, Gondar, Ethiopia; 3 Department of Pediatrics and Child Health Nursing, School of Nursing, College of Medicine and Health Sciences, University of Gondar, Gondar, Ethiopia

**Keywords:** Attitude, First aid, Knowledge, Practice, Teachers, Gondar city

## Abstract

**Background:**

Injuries continue to be an important cause of morbidity and mortality in the developed and developing world. School-age children are more likely to experience unintentional injuries in the school, while they are playing and teachers are the primarily responsible body for keeping the welfare of the students. Knowing the knowledge, attitude, and practice of kindergarten and elementary school teachers towards first aid will be used as an input for policymakers to intervene and provide training. Therefore, this study was aimed to assess knowledge, attitude, practice, and associated factors towards first aid among kindergarten and elementary school teachers in Gondar city, Northwest Ethiopia, 2021.

**Methods:**

An institution-based cross-sectional study was conducted from January 01 to 20, 2021. A simple random sampling technique was employed to recruit 346 participants. A structured pretested self-administered questionnaire was used to collect data. Data were entered in Epi-info version 7, analyzed using SPSS version 21, and presented by frequencies, percentages, tables, and graphs. Bivariable relationships between the independent and outcome variable were investigated using a binary logistic regression model and a multivariable analysis was run to control potential confounding factors. Variables with a *p*-value < 0.05 were considered as factors significantly associated and the strength of association was determined using an odds ratio with a 95% CI.

**Results:**

Only 41.1% of the teachers had good knowledge of first aid. Nearly two-thirds (64.8%) of the teachers had a favorable attitude towards first aid. The majority (85.8%) of the teachers who faced a child in need of first aid in their school gave first aid. Factors like working experience [AOR: 2.45; 95% CI (1.26, 4.73)], school level [AOR: 4.72; 95% CI (1.96, 11.4)], school type [AOR: 4.23; 95% CI (2.07, 8.64)], and having information about first aid [AOR: 2.09; 95% CI (1.11, 3.92)] were significantly associated with knowledge. School-level [AOR = 5.4, 95% CI (2.18–11.67)], school type [AOR = 0.45, 95% CI (0.21–0.94)], and working experience [AOR = 0.33, 95% CI (0.13–0.86)] were the factors significantly associated with attitude.

**Conclusion:**

Less than half and nearly two-thirds of the teachers had good knowledge and a favorable attitude towards first aid. The majority of the teachers who encountered a child in need of first aid gave first aid. Having higher working experience, working in elementary and private schools, and having previous information increases the odds of having good knowledge. Teachers who work in elementary and private schools and have the lower working experience had higher odds of favorable attitude towards first aid. It is better to give attention to the training of staff on first aid specifically for teachers working in kindergarten and governmental schools and new employees and consider integrating first aid in teachers’ training curriculum.

## Background

First aid can be defined as the immediate care given to a person who has been injured or suddenly ill with materials available on hand to preserve life, alleviate suffering, prevent further illness or injury, and promote recovery [1–3]. It is one of the most important procedures to follow in case of a medical emergency or an accident. At least 39% of pre-hospital deaths are potentially preventable with better interventions [4]. An accident is something harmful and takes place suddenly and unexpectedly that may result in simple injuries or major complications like broken bones with heavy bleeding, failure to breathe, unconsciousness, or even death [5]. Many dangerous situations like fainting, falls, intoxications and road crashes that happen at home, school, or in the workplace affect victims and their relatives both physically and psychologically [3].

Unintentional injuries are the leading cause of morbidity and mortality for children [6]. Injuries continue to be an important cause of morbidity and mortality in the developed and developing world [7]. Globally more than 2000 families lost their child due to unintentional injury or accidents every day [5]. The most common causes of accidents and injuries in school children are bullying and assaults, slip and fall accidents, school bus, and playground accidents, food poisoning, and sports activities which result in a significant number of serious injuries [8]. Negligence in injuries or accidents at elementary and kindergarten schools causes the life-threatening condition [9]. The majority of injuries are occurred during free play and on the playground and are precipitated by child-related factors, like being pushed. Boys had significantly higher median injury rates than girls [10].

In Ethiopia, the annual mortality caused by injuries is projected to increase from 10,697 in 2015 to 11,989 by 2030 among children less than 5 years and the number of deaths among 0–14-year olds will be 30,364 [11]. School-age children are more likely to experience unintentional injuries in school, while they are playing. School teachers are the primarily responsible body for keeping the welfare of the students and oversee their activities. They are the first contact and responsible person when children faced injuries. However, studies showed that the level of knowledge and basic practice of first aid among school teachers were found to be poor [12–14].

A study conducted at Lideta sub-city, Addis Ababa and Jimma, Ethiopia among kindergarten teachers, showed that 79.9 and 50.4% of the teachers encountered a child in need of first aid, and 89.7 and 52.1% of teachers gave first aid respectively [13, 14]. In the country, schools have not Emergency Medical Technician (EMT), paramedics, or other trained health professionals who will give first aid. However, pre-hospital school-based Emergency Medical Service (EMS) at school by school personnel is mandatory for saving the children from disability and death. Therefore, assessing the level of knowledge, attitude, and practice of kindergarten and elementary school teachers on first aid is important to intervene and provide training.

## Methods and materials

### Study design and period

An institution-based cross-sectional study was conducted from January 01 to 20, 2021.

### Study setting

The study was conducted at kindergarten and elementary schools found in Gondar city, Northwest Ethiopia. Gondar city is 727 Km far from Addis Ababa, the capital city of Ethiopia, and 180 km from Bahir Dar, the capital city of Amhara regional state. Gondar city has a total area of 192.3 sq. KM. The city has a total population of 338, 646 peoples with 256,041 people whose age is between 18 and 65 years old in 6 sub-cities and 27 Kebele with a total of 78,772 households. Under the Gondar city administration education office there are 11 secondary, 64 elementary, and 70 kindergarten schools with a total of 2649 teachers.

### Study participants

All kindergarten and elementary school teachers working in Gondar city who were available during the data collection period were included in the study. Those teachers who are seriously ill and attending external training courses off-site during the study period were excluded from the study.

### Sample size determination

The sample size was calculated using the single population proportion formula by taking the estimated proportion of knowledge, attitude, and practice among kindergarten and elementary school teachers: 44% [13], a confidence level of 95%, and a margin of error of 5%. The final sample size was 346 after using a correction formula and adding a 10% non-response rate.

### Sampling technique and procedure

A simple random sampling technique was employed to recruit the required participants for the study. First, we stratified participants into elementary and kindergarten school teachers, and then we allocated the required sample for each stratum proportionally. Finally, study participants were selected from each stratum by simple random sampling.

### Data collection instruments and procedures

Data were collected using a structured pre-tested self-administered questionnaire. The questionnaire contains 43 questions arranged into four sections; the first section contains eight questions regarding the socio-demographic characteristics of the participants, the second section contains 11 questions regarding first aid knowledge of kindergarten and elementary school teachers, the third section contains seven attitude related questions, and the last section contains 17 practice-related questions. The questionnaire was adapted from a similar study done at Addis Ababa, Ethiopia [14]. Data were collected with the help of four trained BSc nurse data collectors and two MSc nurse supervisors. A written guideline was given to the data collectors to assure that every participant received the same directions and information. The anonymity of the participant was kept by informing them not to write their name. The instruments were distributed among the study population, after guarantying their willingness to take part in the study, and then it was collected by the data collectors after completion. During data collection data collectors and supervisors followed the recommended precautions to prevent COVID-19.

### Data processing and analysis

Data clean-up and cross-checking were done before analysis. Checked, cleaned, and coded data were entered into EPI info version 7 and exported to SPSS version 21 for analysis. Descriptive statistics like frequencies, percentages, mean and standard deviation, tables, and figures were used to present data. Bivariable relationships between each independent variable and outcome variable were investigated using a binary logistic regression model. Those independent variables with a *p*-value < 0.2 at the bivariable level were included in multivariable analysis to control potential confounding factors. After adjusting their effect on the outcome variable, those variables with a *p*-value < 0.05 with a 95% confidence interval were regarded as factors significantly associated.

### Data quality assurance

The data collection tool was pretested before the actual data collection time at Bahir Dar city kindergarten and elementary schools, which were not included in the study using 5% of the total sample size. Amendments on the instrument, such as unclear questions and ambiguous words were made accordingly. The pretest was also used to estimate how much time it takes to administer the entire questionnaire. The tool was first developed in the English language and translated to the Amharic language with back translation to English for consistency. The one-day training was given to data collectors and supervisors on the objective of the study, instrument, and data collection procedures by the principal investigators. Supervision was conducted by the principal investigators and supervisors. To ensure data quality, each data collector checked the questionnaire from each study participant for completeness daily. The supervisors and principal investigators reviewed each questionnaire daily and checked for completeness.

## Results

### Socio-demographic characteristics of the respondents

A total of 338 respondents have participated in the study with a 97.7% response rate. Among the total participants, 212 (62.7%) respondents were females. One-third (33.4%) of the respondents were in the age group of above 42 years. Of the total participants, 219 (64.8%) respondents were degree holders. Two hundred thirteen (67.5%) of the respondents were married. Among the total respondents, 103 (30.5%) respondents had greater than 10 years of work experience in teaching. The majority (84.9%) of the respondents were from primary schools and 266 (77.8%) of the respondents were from government schools. Most (71.6%) of the respondents did not take the training in first aid (Table 1).

**Table 1:** Socio-demographic characteristics of kindergarten and elementary school teachers in Gondar city, Northwest Ethiopia, 2021 (*n* = 338)

Variables	Response	Frequency	Percentage (%)
Sex	Male	126	37.3
	Female	212	62.7
Age	22-31 years	108	32.0
	32–41 years	117	34.6
	Above 42 years	113	33.4
Level of education	Certificate	9	2.7
	Diploma	110	32.5
	Degree	219	64.8
Marital status	Married	213	67.5
	Single	101	29.9
	Divorced	15	4.4
	Widowed	9	2.7
Service year	< 10 years	146	43.2
	11–20 years	89	26.3
	> 21 years	103	30.5
School-level	Kindergarten	51	15.1
	Elementary	287	84.9
School type	Government	263	77.8
	Private	75	22.2
Do you have training on first aid	Yes	96	28.4
	No	242	71.6

### Knowledge of kindergarten and elementary school teachers towards first aid

**Fig. 1:**
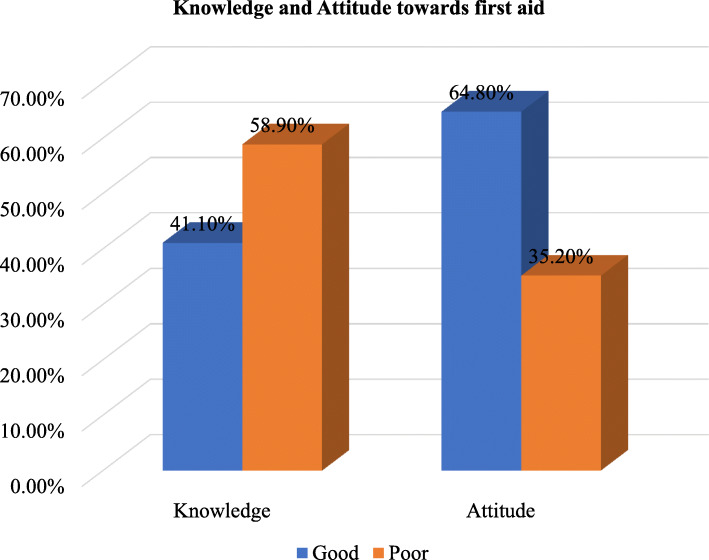
Knowledge and attitude of kindergarten and elementary schools teachers towards first aid in Gondar city, Northwest Ethiopia, 2021 (*n* = 338)

Of the total respondents, only 41.1% (with 95% CI (35.9, 45.7%)) of the respondents had good knowledge of first aid (Fig. 1). The majority (81.7%) of the participants had information about first aid. Of these, 10.9, 7.6, 21.0, and 60.5% were heard from family, books, media, and health professionals respectively. The majority (93.5%) of the respondents correctly respond to what first aid means. Nearly two-thirds (66.3%) of the participants were aware of giving nothing by mouth for fainting children. More than three fourth (79.6%) of the respondents understand the concept of immobilization for neck and back injuries (Table 2).

**Table 2:** Knowledge of kindergarten and elementary schools teachers towards first aid in Gondar city, Northwest Ethiopia, 2021 (*n* = 338)

Item	Responses	
	Yes (%)	No (%)
Have you ever heard about first aid	276 (81.7)	62 (18.3)
First aid is the immediate care given for a person who sustained an injury or accident before the victim arrives health institution	316(93.5)	22(6.5)
Pressing firmly with a clean bandage the bleeding part is one of the measures to stop bleeding	301(89.1)	37(10.9)
Giving fluid by mouth is one of the first aid measures for fainting a child	114(33.7)	224(66.3)
One of the first aid measures for an epileptic child is keeping the airway clear by placing the child on his/her back	229(67.8)	109(32.2)
Standing behind the child encircling the child’s chest by hands and squeezing is the first aid measure for choking a child	240(71.0)	98(29.0)
For a child with neck and back injury avoiding head and neck movement and keeping the body straight is one measure of first aid	269(79.6)	69(20.4)
In case a child has bitten by his friend, cleansing wound with soap and water for 5 min is one measure of first aid for a human bite	233(68.9)	105(31.1)
One of the first aid measures for nose bleed/epistaxis is placing student sitting comfortably with slightly backward and applying uninterrupted pressure by pressing nostrils together	257(76.0)	81(24.0)
Encouraging the child to sit quietly, breathe slowly and deeply in through the nose and out through the mouth is the first aid measure for the child with the difficulty of breathing	292(86.4)	46(13.6)

### The attitude of kindergarten and elementary school teachers towards first aid

Among the total participants, nearly two-thirds (64.8%) with 95% CI (59.2–69.8%) of the respondents had a favorable attitude towards first aid (Fig. 1). About 200 (59.2%) of the respondents strongly agreed with the idea giving first aid at school is fair. One hundred three (30.5%) of the respondents were strongly disagreed with giving first aid at school is unpleasant. About half (49.4%) of the respondents were strongly disagreed with giving first aid is not good. About 207 (61.2%) of the respondents were strongly agreed with giving special care for injured children in academic work is appropriate (Table 3).

**Table 3:** Attitude of kindergarten and elementary school teachers towards first aid in Gondar city, Northwest Ethiopia, 2021 (*n* = 338)

Item	Response			
	Strongly disagree	Disagree	Agree	Strongly agree
	Frequency (%)	Frequency (%)	Frequency (%)	Frequency (%)
Giving first aid at school is fair	35 (10.4)	20 (5.9)	83 (24.3)	200 (59.2)
Giving first aid at school is unpleasant	103 (30.5)	167 (49.4)	49 (14.5)	19 (5.6)
Giving first aid is not good	167 (49.4)	129 (38.2)	28 (8.3)	14 (4.1)
It is good for me to provide first aid	26 (7.7)	23 (6.8)	97 (28.7)	192 (56.8)
I need to learn first aid	29 (8.6)	21 (6.2)	85 (25.1)	203 (60.1)
It is the responsibility of the teacher to giving first aid care for children in need	26 (7.7)	62 (18.3)	169 (50.0)	81 (24.0)
Giving special care for injured children in academic work is appropriate	23 (6.8)	22 (6.5)	86 (25.4)	207 (61.2)

### The practice of kindergarten and elementary school teachers towards first aid

More than three-fourths (76.9%) of the teachers faced a child in need of first aid in their school and 85.8% of them gave first aid for the child. Ninety-five (28.1%) of the participants faced a child with difficulty of breathing and nearly half (49.5%) of them encouraged the student to sit quietly. One hundred forty-six (43.2%) of the respondents faced a child with fainting and 75.3% of them kept the student in a flat position. Nearly three-fourths (74.9%) of the teachers faced a child with bleeding from his/her nose and more than half (51.0%) of them applied uninterrupted pressure by pressing nostrils together. Nearly half (50.6%) of the participants faced a child with bleeding on his/her body and 35.1% of them pressed firmly with a clean bandage to stop bleeding. One hundred forty-eight (43.8%) of the teachers faced a child with seizure/epilepsy and 41.2% of them moved surrounding objects to avoid injury. Nearly one-third of the respondents faced a child with choking and more than half (58.3%) of them stood behind the child encircling the child’s chest by hands and squeezed. Nearly one-fifth (19.5%) of the participants faced a child with an injured neck and back and more than half (54.6%) of them avoided head and neck movement and kept their body straight (Table 4).

**Table 4:** Practice of kindergarten and elementary school teachers towards first aid in Gondar city, Northwest Ethiopia, 2021 (n = 338)

Item	Response	Frequency (percentage)
Have you ever faced a child in need of first aid in your school?	Yes	260 (76.9%)
	No	78 (23.1%)
If yes, did you give first aid (*n* = 260)?	Yes	223 (85.8%)
	No	37 (14.2%)
Have you ever faced a child with difficulty of breathing?	Yes	95 (28.1%)
	No	243 (71.9%)
If yes, what did you do (*n* = 95)?	Called ambulance	33 (34.7%)
	Encouraged the student to sit quietly	47 (49.5%)
	Encouraged slow and deep breath in through the nose and out through the mouth	15 (15.8%)
Have you ever faced a child with fainting?	Yes	146 (43.2%)
	No	192 (56.8%)
If yes, what did you do (*n* = 146)?	Called ambulance	15 (10.3%)
	Kept the student in a flat position	110 (75.3%)
	Loosen clothing around the neck and waist	21 (14.4%)
Have you ever faced a child with bleeding from his/her nose?	Yes	253 (74.9%)
	No	85 (25.1%)
If yes, what did you do (*n* = 253)?	Called ambulance	21 (8.3%)
	Laid on the side with head raised on a pillow	57 (22.5%)
	Applied uninterrupted pressure by pressing nostrils together	129 (51.0%)
	Applied ice to nose	46 (18.2%)
Have you ever faced a child with bleeding on his/her body?	Yes	171 (50.6%)
	No	167 (49.4%)
If yes, what did you do (*n* = 171)?	Called ambulance	18 (10.5%)
	Pressed firmly with a clean bandage to stop bleeding	60 (35.1%)
	Elevated bleeding body part gently	50 (29.2%)
	Bandaged bleeding wound without interfering circulation	43 (25.2%)
Have you ever faced a child with seizure/epilepsy?	Yes	148 (43.8%)
	No	190 (56.2%)
If yes, what did you do (*n* = 148)?	Called ambulance	6 (4.1%)
	Placed on the floor	53 (35.8%)
	Moved surrounding objects to avoid injury	61 (41.2%)
	Avoided giving any drink or food by mouth	28 (18.9%)
Have you ever faced a child with choking?	Yes	108 (32.0%)
	No	230 (68.0%)
If yes, what did you do (*n* = 108)?	Called ambulance	6 (5.6%)
	Stood behind the child encircling the child’s chest by hands and squeezed	63 (58.3%)
	Continued squeezing until the object expelled	39 (36.1%)
Have you ever faced a child with an injured neck and back?	Yes	66 (19.5%)
	No	272 (80.5%)
If yes, what did you do (*n* = 66)?	Called ambulance	9 (13.6%)
	Laid the student and restrict moving unless harm exacerbated if the students stayed there	21 (31.8%)
	Avoided head and neck movement and kept body straight	36 (54.6%)

### Factors associated with knowledge of teachers towards first aid

Using bivariable logistic regression analysis age, experience, school level, school type, training, having information about first aid, and history of exposure with a child in need of first aid were found to be significantly associated with knowledge. In multivariable logistic regression analysis, working experience, school level, school type, and having information about first aid were significantly associated with the knowledge of teachers towards first aid.

Teachers who worked for 11–20 years were nearly three times higher to be knowledgeable compared with teachers who worked for less than 10 years [AOR: 2.45; 95% CI (1.26, 4.73)]. Those teachers working at elementary schools were nearly five times higher to have good knowledge compared with teachers working at kindergarten schools [AOR: 4.72; 95% CI (1.96, 11.4)]. Those study participants who work at private schools were nearly four times higher to have good knowledge about first aid compared with governmental school teachers [AOR: 4.23; 95% CI (2.07, 8.64)]. Those teachers who had information about first aid were two times higher to have good knowledge compared with their counterparts [AOR: 2.09; 95% CI (1.11, 3.92)] (Table 5).

**Table 5:** Bivariable and multivariable logistic regression analysis of factors associated with knowledge towards first aid among kindergarten and elementary school teachers in Gondar city, Northwest Ethiopia, 2021 (*n* = 338)

Variables		Knowledge		OR with 95% CI		*P*-value
		Good	Poor	Crude	Adjusted	
Age	> = 42	58	55	2.30(1.33,3.97)	2.20(0.64, 7.60)	0.213
	32–41	47	70	1.46(0.84, 2.53)	1.35(0.63, 2.91)	0.441
	22–31	34	74	1	1	
Experience	> = 20	48	55	1.90(1.13, 3.20)	1.81(0.96, 3.43)	0.068
	11–20	45	44	2.22(1.30, 3.83)	2.45(1.26, 4.73)	0.008*
	<=10	46	100	1	1	
School-level	Elementary Kindergarten	129	15,841	3.35(1.61, 6.94)	4.72(1.96, 11.40)	0.001*
		10		1	1	
School type	Private Governmental	40	35	1.89(1.13, 3.18)	4.23 (2.07, 8.64)	< 0.001*
		99	164	1	1	
Training	Yes	51	45	1.98(1.23, 3.20)	1.28(0.75, 2.18)	0.338
	No	88	154	1	1	
Having previous	Yes	121	155	1.91(1.05, 3.47)	2.09 (1.11, 3.92)	0.023*
Information	No	18	44	1	1	
Previous experience of situations requiring first aid	Yes	120	140	2.66(1.50, 4.71)	1.80(0.97, 3.34)	0.065
	No	19	59	1	1	

* Statistically significant at *p*-value < 0.05

### Factors associated with the attitude of teachers towards first aid

Bivariable and multivariable logistic regression analyses were carried out. Age, training, school level, school type, and experience were eligible for multivariable analysis. In multivariable analysis, the factors significantly associated with the attitude of the participants were; school level, school type, and experience. Teachers who work in elementary schools were five times more likely to have a favorable attitude towards first aid than teachers who work in kindergarten [AOR = 5.4, 95% CI (2.18–11.67)]. Teachers who work in governmental schools were 55% times less likely to have a favorable attitude towards first aid than teachers who work in private schools [AOR = 0.45, 95% CI (0.21–0.94)]. Teachers who work for greater than equal to 21 years were 67% times less likely to have a favorable attitude towards first aid than teachers who work for ten and below 10 years [AOR = 0.33, 95% CI (0.13–0.86)] (Table 6).

**Table 6:** Bivariable and multivariable logistic regression analysis of factors associated with the attitude of teachers towards first aid in Gondar city, Northwest Ethiopia, 2021 (n = 338)

Variables		Attitude		COR (95%CI)	AOR (95%CI)	***P***-value
		Good	Poor			
**Age**	22–31	67	41	1.0	1.0	
	32–41	79	38	1.27 (0.73–2.20)	1.07 (0.48–2.38)	0.855
	> = 42	73	40	1.11(0.64–1.93)	0.39 (0.11–1.33)	0.133
**Training**	Yes	91	68	0.68 (0.41–1.14)	0.81(0.47–1.41)	0.467
	No	151	28	1.0	1.0	
**School-level**	Kindergarten	26	25	1.0	1.0	
	Elementary	193	94	1.97 (1.08–3.60)	5.4 (2.18–11.67)	0.001*
**School type**	Government	169	94	0.89 (0.52–1.54)	0.45 (0.21–0.94)	0.034*
	Private	50	25	1.0	1.0	
**Experience**	< = 10	93	53	1.0	1.0	
	11–20	56	33	0.96 (0.56–1.67)	0.37 (0.16–1.23)	0.106
	> = 21	70	33	1.2 (0.71–2.06)	0.33 (0.13–0.86)	0.024*

* Statistically significant at *P*-value < 0.05

## Discussion

The result of this study revealed that only 41.1% of the respondents have good knowledge of first aid. The finding of this study is in line with previous studies conducted in Addis Ababa, 40.0% [14], Debretabor, 45.8% [12], and Jimma, Ethiopia 44.4% [13]. According to the participant’s response, 85.8% of the respondents report as they give first aid for injuries and illness however less than half of the participants have good knowledge. It implies that some of the teachers exercise first aid interventions without basic knowledge. It shall be given attention and first aid training to equip school teachers with first aid knowledge to give evidence-based first aid for accidents.

The finding of this study was higher than a study conducted in Saudi Khamis mushyt city, 19.6% [15]. The difference might be due to variation in sample size and socio-demographic characteristics of the respondents. On the other hand, the result of this study was lower than the studies conducted in Malaysia 77.4% [16] and Iraq 95% [17]. The possible reason for this variation might be due to differences in a school setup, socio-demographic characteristics of the respondents, and variation in the measurement. The previous studies were conducted among elementary school teachers whereas the current study was conducted among both elementary and kindergarten school teachers.

This study revealed that 64.8% of kindergarten and elementary school teachers had a favorable attitude towards first aid. This finding was in line with a study conducted in Saud Arabia (67%) [18] and Riyadh (68.4%) [19]. This might be due to the similarities of teachers in academic activities in the school. However, this finding was lower than studies conducted in Debre tabor, Ethiopia (75%) [12], Addis Ababa, Ethiopia (75%) [14], and Jatinangor (71.5%) [20]. This discrepancy might be due to the variation of teacher’s academic performance, knowledge, training about first aid, and the school settings across those areas. The previous study conducted in Debre tabor was conducted only among elementary school teachers and the study in Addis Ababa, Ethiopia was conducted among kindergarten school teachers only. On the other hand, our study incorporates the attitude of both elementary and kindergarten school teachers.

In the current study, 85.8% of the teachers who encountered children in need of first aid gave first aid to the child. This finding was relatively consistent with a study conducted in Addis Ababa, Ethiopia (89.7%) [14]. However, it was higher than studies conducted in Debre Tabor, Ethiopia (64%), Jimma, Ethiopia (52.1%), Khamis Mushayt City, Saudi Arabia (54.9%), and Indonesia (78.8%) [12, 13, 15, 20]. The possible justification for this difference might be due to the difference in data collection tool used in each study, study participants, and knowledge level. The current study was conducted among kindergarten and elementary school teachers whereas the previous studies were conducted among either kindergarten or elementary school teachers only.

Working experience, school level, school type, and having information about first aid were significantly associated with knowledge towards first aid. The odds of having good knowledge were nearly three times higher among teachers who had 11–20 years of experience compared with those teachers who had less than 10 years of experience. This finding was supported by studies conducted in Debre tabor, Ethiopia, Addis Ababa, Nigeria, Malaysia, Al-Qassim Saudi Arabia, and Khamis Mushyt city Saudi Arabia [12, 14–16, 21, 22]. This might be due to learning from experience as they faced individuals in need of first aid, training on first aid, and pre-hospital service. The finding of this study revealed that special consideration should be given to the newly employed teachers. This might also be due to as teacher’s working experience increases their academic studies will be complemented by another way of learning from day-to-day exposure. In turn, previous exposure of a child in need of first aid also provides them with crucial knowledge, skills, and personal attributes through communication, team-working and problem-solving skills.

The odds of having good knowledge were five times higher among elementary school teachers compared with kindergarten school teachers. It might be due to the difference in the level of education. According to the result of this study, almost all of the elementary school teachers were diploma and degree holders while kindergarten school teachers were certificate. This implies that special attention should be given to kindergarten school teachers since they give care for kids who didn’t aware of their environment and susceptible to accidents. Private school teachers were four times higher to be knowledgeable compared with teachers who work at governmental schools. This might be due to the difference in a school setup. Most of the time private schools are business-oriented, competitive, and well equipped with infrastructures including first aid kits. To attract customers and to be competitive private schools might provide training for teachers about first aid and accident prevention which makes private school teachers more knowledgeable compared with governmental school teachers.

Similarly, Participants who had previous information about first aid were two times higher to be knowledgeable compared with their counterparts. This finding is supported by studies conducted in Addis Ababa [20] and Debretabor, Ethiopia [12]. This might be due to having previous information regarding first aid leads to a higher score of knowledge-related questions than respondents who didn’t have information about the issue. This implies that obtaining information about first aid and emergency medical care either from media, training, health professionals, or family increases the acquisition of knowledge towards first aid.

School-level, school type, and working experience were significantly associated with the attitude towards first aid. Teachers who work in elementary schools were five times more likely to have a favorable attitude towards first aid compared with teachers who work in kindergarten schools. This might be due to those teachers who work in primary schools were more knowledgeable about first aid since the school level determines the teacher’s competency. Governmental school teachers were 55% times less likely to have a favorable attitude towards first aid than teachers who work in private schools. This might be due to private schools might have good standards and structures of the school including first aid kits and their teachers also might have strict control since private schools are business-oriented. Similarly, the working experience was significantly associated with the attitude of teachers towards first aid. Teachers who had working experience of greater than or equal to 21 years were 67% times less likely to have a favorable attitude compared with teachers who had working experience of less than or equal to 10 years. This finding was supported by other studies [14, 23], [24]. This might be due to those teachers with long time experience give less attention to first aid because mostly they are old age and their academic status is diploma whereas younger age groups of teachers were degree holders.

### This study has some limitations

There might be a possibility of social desirability and recall bias. We were unable to identify factors associated with practice due to the variation in the type of cases requiring first aid. The generalizability of the findings to schools in other parts of the country might be compromised since the study was conducted in one city. We were also unable to make an adequate comparison with other studies due to the lack of similar studies.

## Conclusion

Less than half and nearly two-thirds of the teachers had good knowledge and a favorable attitude towards first aid. The majority of the teachers who encountered a child in need of first aid gave first aid. Having higher working experience, working in elementary and private schools, and having previous information about first aid increases the odds of having good knowledge of first aid. Teachers who work in elementary and private schools and have the lower working experience had higher odds of favorable attitude towards first aid. It is better to give attention to the training of staff on first aid specifically for teachers working in kindergarten and governmental schools and new employees. Policymakers in education might use the finding of this study as an input to integrate first aid in teachers’ training curriculum.

## Data Availability

The datasets used and/or analyzed during the current study available from the corresponding author on reasonable request.

## Abbreviations

AOR: Adjusted odds ratio
CI: Confidence interval
EMS: Emergency medical service
EMT: Emergency medical technician
SPSS: Statistical package for social sciences
